# Stromal derived factor 1α: A chemokine that delivers a two-pronged defence of the myocardium^☆^

**DOI:** 10.1016/j.pharmthera.2014.03.009

**Published:** 2014-09

**Authors:** Daniel I. Bromage, Sean M. Davidson, Derek M. Yellon

**Affiliations:** The Hatter Cardiovascular Institute, 67 Chenies Mews, London WC1E 6HX, United Kingdom

**Keywords:** SDF, DPP-4, Cardioprotection, AMI, Ischemic conditioning, CXCR4

## Abstract

Alleviating myocardial injury associated with ST elevation myocardial infarction is central to improving the global burden of coronary heart disease. The chemokine stromal cell-derived factor 1α (SDF-1α) has dual potential benefit in this regard. Firstly, SDF-1α is up-regulated in experimental and clinical studies of acute myocardial infarction (AMI) and regulates stem cell migration to sites of injury. SDF-1α delivery to the myocardium after AMI is associated with improved stem cell homing, angiogenesis, and left ventricular function in animal models, and improvements in heart failure and quality of life in humans. Secondly, SDF-1α may have a role in remote ischaemic conditioning (RIC), the phenomenon whereby non-lethal ischaemia–reperfusion applied to an organ or tissue remote from the heart protects the myocardium from lethal ischaemia–reperfusion injury (IRI). SDF-1α is increased in the serum of rats subjected to RIC and protects against myocardial IRI in ex vivo studies. Despite these potential pleiotropic effects, a limitation of SDF-1α is its short plasma half-life due to cleavage by dipeptidyl peptidase-4 (DPP-4). However, DPP-4 inhibitors increase the half-life of SDF-1α by preventing its degradation and are also protective against lethal IRI. In summary, SDF-1 potentially delivers a ‘two-pronged’ defence of the myocardium: acutely protecting it from IRI while simultaneously stimulating repair by recruiting stem cells to the site of injury. In this article we examine the evidence for acute and chronic cardioprotective roles of SDF-1α and discuss potential therapeutic manipulations of this mechanism with DPP-4 inhibitors to protect against lethal tissue injury in the clinical setting.

## 1 Introduction

Coronary heart disease is the leading cause of death worldwide, accounting for an estimated 7.3 million deaths per year (“Global Atlas on Cardiovascular Disease Prevention and Control”). Untreated, mortality following ST-elevation myocardial infarction (STEMI) may be as high as 15% and strategies to mitigate the deleterious effects of STEMI are therefore paramount (Gibson, 2004). Early reperfusion by primary percutaneous coronary intervention (PPCI) is the most effective strategy for reducing infarct size and improving clinical outcome (Keeley et al., 2003, Gibson, 2004). Other important therapeutic targets include platelet aggregation, subsequent myocardial dysfunction and secondary prevention, including statin therapy. Overall, 30 day mortality following PPCI in the UK is now 6.5% (“BCIS Audit Returns 2012: Adult Interventional Procedures”). Another potential target is the injury inflicted by the therapeutic restoration of blood flow, known as ischaemia–reperfusion injury (IRI), which may account for up to 50% of final infarct size (Braunwald and Kloner, 1985, Piper et al., 1998, Staat et al., 2005, Yellon and Hausenloy, 2007). The chemokine stromal cell-derived factor 1α (SDF-1α) potentially delivers a ‘two-pronged’ defence of the myocardium in this regard: acutely protecting the myocardium from IRI while simultaneously stimulating myocardial repair by recruiting stem cells to the site of injury.

SDF-1α is known to play a central role in stem cell homing, retention, survival, proliferation, cardiomyocyte repair, angiogenesis and ventricular remodelling following myocardial infarction (Kucia, Dawn, et al., 2004, Cheng et al., 2008, Saxena et al., 2008, Tang et al., 2009, Zaruba et al., 2009, Jujo et al., 2010, Takahashi, 2010, Tang et al., 2010, Zaruba and Franz, 2010, Ghadge et al., 2011, Kanki et al., 2011, Dong et al., 2012, Penn et al., 2012). It acts as the unique ligand for its receptor CXCR4 and the SDF-1–CXCR4 axis is up-regulated in both experimental and clinical studies of myocardial infarction (Zaruba & Franz, 2010). SDF-1α–CXCR4 has been utilised to target stem cells to ischaemic tissue, thereby improving left ventricular (LV) dimensions and function (Misao et al., 2006, Sasaki et al., 2007, Saxena et al., 2008, Tang et al., 2010). Importantly, the SDF-1α–CXCR4 signalling axis exerts these effects via a Gα1 dependent mechanism and activation of phosphoinositide 3 kinase (PI3K), mitogen activated protein kinase (MAPK), and Janus kinase (JAK)-signal transducer and activator of transcription (STAT) signalling.

These signalling pathways are the same pathways that it is postulated are responsible for the protection against IRI conferred by all forms of conditioning such as pre, post and remote ischaemic conditioning (Hausenloy and Yellon, 2004, Hausenloy and Yellon, 2007a, Hausenloy and Yellon, 2007b). The latter describes the phenomenon whereby non-lethal ischaemia and reperfusion applied to an organ or tissue remote from the heart protects the myocardium from lethal reperfusion injury (Przyklenk et al., 1993, Whittaker and Przyklenk, 1994, Dickson et al., 2000, Hausenloy and Yellon, 2008a, Hausenloy and Yellon, 2008b). Remote ischaemic conditioning (RIC) can be induced non-invasively by inflating a blood pressure cuff placed on the arm or thigh to above systolic pressure to induce brief ischaemia and then deflating the cuff to allow reperfusion (Kharbanda et al., 2002). When administered pre-hospital this has been shown to reduce myocardial infarct size and improve myocardial salvage in PPCI patients (Botker et al., 2010), and improve outcomes in patients undergoing cardiac surgery or elective PCI (Gunaydin et al., 2000, Hausenloy et al., 2007, Hoole et al., 2009). The mechanism of cardioprotection conferred by RIC is so far unknown, but is thought to be due to a humoral factor that has been shown by biochemical fractionation studies to be a protein between 3.5 kDa and 15 kDa in size (Serejo et al., 2007, Shimizu et al., 2009).

**Fig. 1:**
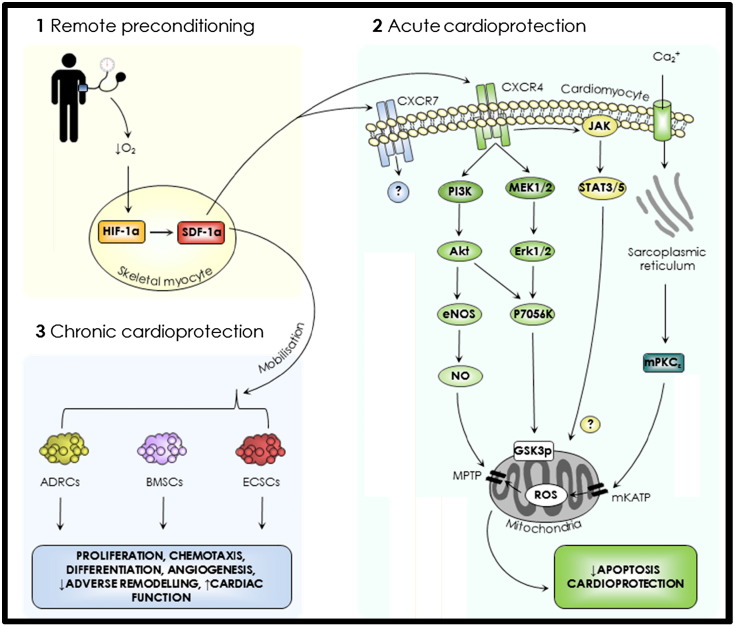
The SDF-1–CXCR4 signalling axis. Remote preconditioning may enable both acute and chronic cardioprotective pathways (see text for details). SDF-1α, stromal derived factor-1α; HIF-1α, hypoxic inducible factor-1α; ADRCs, adipose tissue derived regenerative cells; BMSCs, bone marrow stem cells (including mesenchymal stem cells, hamatopoietic stem cells and endothelial stem cells); ECSCs, endogenous cardiac stem cells; JAK, Janus kinase; STAT, signal transducer and activator of transcription; PI3K, phosphoinositide 3 kinase; MEK1/2, mitogen-activated protein kinase; Erk, extracellular signal-regulated kinases; eNOS, endothelial nitric oxide synthase; NO, nitric oxide; mPKC, mitochondrial protein kinase C; mK_ATP_, mitochondrial ATP-sensitive potassium channel; ROS, reactive oxygen species; MPTP, mitochondrial permeability transition pore.

It is therefore proposed that in addition to its chronic effects SDF-1α has a direct role in the protection observed from RIC and a proposed paradigm is described in Fig. 1 (Saxena et al., 2008, Zaruba and Franz, 2010, Huang et al., 2011). Recently published data has demonstrated increased SDF-1α in the serum of rats subjected to RIC. Furthermore, RIC, which was shown to significantly decrease infarct size and improve papillary muscle functional recovery in an ex vivo model, could be blocked by AMD3100, a highly specific inhibitor of CXCR4 (Davidson et al., 2013, De Clercq, 2003). This, together with the identification of SDF-1α as an 8 kDa peptide has made it a prime candidate for a role in RIC (Ceradini et al., 2004). In this review we focus on the evidence for acute and chronic roles of SDF-1α in cardioprotection following STEMI and discuss potential therapeutic manipulations of this mechanism to protect against lethal tissue injury in the clinical setting. In particular we examine the role of inhibitors of the protease dipeptidyl peptidase 4 (DPP-4) to prolong the half-life of SDF-1α to these ends.

## 2 Stromal derived factor 1α–CXC Chemokine Receptor 4/CXC Chemokine Receptor 7

Chemokines, or chemoattractant cytokines, play a critical role in the regulation and trafficking of leukocytes as well as haematopoietic and other progenitor cells (Ghadge et al., 2011). They are also involved in a variety of other functions, including degranulation, mitogenesis, gene transcription, apoptosis, and angiogenesis (Gerard & Rollins, 2001). There are over 50 human chemokines that are classified according to the position of two N-terminal cysteine residues as CXC, CCC, C or CX3C (Ghadge et al., 2011). In myocardial ischaemia, several chemokines, including CXC and CC subtypes, have been shown to be up-regulated in both experimental and clinical studies of myocardial infarction (Matsumori et al., 1997, Riesenberg et al., 1997, Frangogiannis and Entman, 2005, Takahashi, 2010, Ghadge et al., 2011).

SDF-1 (also known as CXCL12) is a CXC chemokine, so-called because the two amino acids nearest the N-terminus are separated by a single amino acid (Rollins, 1997). It is a small (8 kDa), 89 amino acid peptide that is encoded by a gene originally cloned from murine bone marrow stromal cells, hence its name (Rollins, 1997, Ceradini et al., 2004, Ghadge et al., 2011). It is highly conserved (more than 92% similarity at the protein level) between species and has been shown to confer protection between species (Shirozu et al., 1995, Shimizu et al., 2009). Several isoforms of SDF-1, namely SDF-1α–ζ, arise from alternative splicing and have been identified by PCR (Ghadge et al., 2011). Of interest here and best described is SDF-1α. Note that many studies do not specify which isoform they are investigating and henceforth where this is the case the more general SDF-1 descriptor is adopted. SDF-1α expression has been reported in several organs, tissues and cells, including bone marrow, heart, liver, kidney, thymus, spleen, skeletal muscle, brain and, more recently, platelets (Kucia et al., 2005, Nagasawa, Nakajima, et al., 1996, Ratajczak et al., 2006, Ghadge et al., 2011, Chatterjee and Gawaz, 2013), although it is likely that this also reflects expression in the vascular endothelium of the organ. In the heart, SDF-1α is expressed by stromal cells, endothelial cells and cardiomyocytes (Askari et al., 2003, Segret et al., 2007). It is a chemoattractant for a variety of cell types including T lymphocytes, bone marrow stem cells (BMSCs; including haematopoietic, endothelial and mesenchymal subtypes), adipose-derived regenerative cells (ADRCs) and c-kit^+^ endogenous cardiac stem cells (eCSCs) (Aiuti et al., 1997, Rollins, 1997, Kondo et al., 2009, Zaruba and Franz, 2010). It also has a role in maintaining hematopoietic stem cell niches in bone marrow (Ghadge et al., 2011). SDF-1α is cleaved from its active form (1–68) to SDF-1α (3–68) by exopeptidases such as dipeptidyl peptidase 4 (DPP-4), matrix metalloproteinase (MMP)-2 and MMP-9 (Segers et al., 2007). It has an estimated half-life in vivo by radiolabelling of 25.8 ± 4.6 min (Misra et al., 2008). However, this represents total SDF-1α only and does not differentiate active and inactive isoforms (Misra et al., 2008). Similarly, commercially available ELISA kits, to our knowledge, measure total and not active SDF-1α only and may therefore offer a skewed view of the role of SDF-1α in myocardial infarction.

SDF-1α exerts its effects by binding the receptors CXCR7 and CXCR4, for which it is the unique ligand (Balabanian et al., 2005, Sierro et al., 2007). CXCR4 is a G protein coupled receptor that, once activated, is thought to initiate a signalling cascade that regulates the functions described (Kucia, Jankowski, et al., 2004, Wong and Korz, 2008, Ghadge et al., 2011). The SDF-1α–CXCR4 axis is also important in embryogenesis, including cell migration and development of neuronal, cardiac, vascular, haematopoietic and craniofacial systems, which is reflected by its expression on a range of progenitor cells, including haematopoietic, endothelial and cardiac stem cells (Rollins, 1997, Zaruba and Franz, 2010, Ghadge et al., 2011). During angiogenesis, expression of CXCR4 on vessel endothelium correlates with areas of high SDF-1α expression (McGrath et al., 1999). In embryonic development transgenic homozygote mice lacking either CXCR4 or SDF-1α have abnormal B-lymphocyte development, as well as abnormal hepatic and cardiac development, including ventricular septal defects, and die in utero (Nagasawa, Hirota, et al., 1996, Tachibana et al., 1998, Zou et al., 1998). Similarly, in humans, CXCR4 mutation causes impaired mobilisation of neutrophils and B-cell lymphopaenia (Hernandez et al., 2003).

More recently it has become apparent that CXCR7 also plays a central role in SDF-1α regulation. For example, Berahovich et al. found increased serum SDF-1α in a murine model of genetic deletion and pharmacological inhibition of CXCR7, which was associated with impaired leukocyte migration (Berahovich et al., 2014). However, the relative contribution and relationship of CXCR4 and CXCR7 is not fully elucidated. Hoffman et al. have demonstrated the rapid spontaneous internalisation of SDF-1α associated with CXCR7, compared to G-protein coupling via CXCR4, and rapid release of SDF-1α degradation products (Hoffmann et al., 2012). They also described a mechanism whereby CXCR7 performs an SDF-1α-scavenging function (Hoffmann et al., 2012). CXCR7 is thought to be up-regulated in inflammation, cancer and autoimmune conditions (Sanchez-Martin et al., 2013). It has been shown to improve migration and paracrine (angiogenesis and mitogenesis) function of mesenchymal stem cells (MSCs) in mice subjected to renal IR, and also reduced apoptosis and improved functional recovery (Liu et al., 2012). Despite this its contribution, if any, to cardioprotection is not yet known (Zaruba & Franz, 2010).

## 3 Chronic cardioprotection

### 3.1 Stromal derived factor 1α–CXC Chemokine Receptor 4/CXC Chemokine Receptor 7 after acute myocardial infarction

Much is known about the SDF-1α–CXCR4/CXCR7 axis in the context of myocardial regeneration after acute myocardial infarction (‘chronic cardioprotection’). The mechanism is well described. In hypoxic conditions, the transcription factor HIF-1α is up-regulated and in turn up-regulates SDF-1α, CXCR4 and CXCR7 expression (Pillarisetti and Gupta, 2001, Zaruba and Franz, 2010, Ghadge et al., 2011, Esencay et al., 2013). In this way SDF-1α acts as diffusible ‘homing beacon’ directing cells towards hypoxic tissue. In health, since the bone marrow is physiologically hypoxic, SDF-1α expression results in the retention of bone marrow stem cells (Ceradini et al., 2004, Zaruba and Franz, 2010). In response to acute myocardial infarction (AMI) several clinical studies have demonstrated up-regulation of SDF-1α in infarct and peri-infarct zones, returning to baseline at 7 days (Pillarisetti and Gupta, 2001, Askari and Penn, 2003, Abbott et al., 2004, Hu et al., 2007), and in the serum of patients suffering AMI (Yamani et al., 2005, Leone et al., 2006). SDF-1α–CXCR4 signalling is also reportedly elevated in toxic liver damage, total body irradiation and after chemotherapy (Kucia et al., 2005). In AMI, this transient increase causes the gradient-guided homing of progenitor cells from the bone marrow, as well as adipose and cardiac tissue, to sites of injury and inflammation. Thus, expression of SDF-1 in infarcted myocardium has been associated with recruitment, retention, survival and proliferation of ADRCs, eCSCs and BMSCs (Kucia, Dawn, et al., 2004, Unzek et al., 2007, Kondo et al., 2009, Jujo et al., 2010, Tang et al., 2010, Zaruba and Franz, 2010, Penn et al., 2012). Conversely, decreased recruitment, angiogenesis and blood flow has been demonstrated when CXCR4 is blocked on infused progenitor cells or SDF-1α is blocked in the recipient (Ceradini et al., 2004, Zaruba and Franz, 2010). SDF-1α–CXCR4/CXCR7 exerts these effects by activating intracellular signalling cascades (Zaruba & Franz, 2010). These include the MAPK p42/44 extracellular signal-regulated (Erk1/2), PI3K-Akt, JAK-STAT and protein kinase C (PKC) signalling cascades, as well as inositol-1,4,5-triphosphate (IP3)-induced SR/ER calcium release (Ratajczak et al., 2006, Haider et al., 2008, Gao et al., 2009, Zaruba and Franz, 2010, Ghadge et al., 2011).

Although a number of mechanisms can mobilise stem cells, including cytokines (granulocyte- and granulocyte macrophage-colony stimulating factor (G-CSF, GM-CSF), and stem cell factor (SCF)), interleukins (IL-7, IL-12, IL-3), chemokines (SDF-1α, IL-8), growth factors (VEGF, hepatocyte- and insulin-like growth factor (HGF, IGF)) (Lapidot & Petit, 2002), and chemotherapeutic agents like cyclophosphamide (Ghadge et al., 2011), it is suggested that the SDF-1α–CXCR4 axis is the most potent and central to the process of mobilising progenitor cells (Lapidot and Petit, 2002, Cottler-Fox et al., 2003). For example, G-CSF, a cytokine known to increase the mobilisation of stem cells from bone marrow and widely used therapeutically, does so by disrupting the association of SDF-1 on bone marrow stromal cells, osteoblasts and reticulocytes with CXCR4 on bone marrow stem cells (Petit et al., 2002, Levesque et al., 2003, Zaruba and Franz, 2010). Interestingly, a similar mechanism may explain how AMD3100, a selective CXCR4 antagonist, increases circulating HPCs and endothelial progenitor cells (Liles et al., 2003, Jujo et al., 2010).

### 3.2 Therapeutic application of stromal derived factor 1α–CXC Chemokine Receptor 4/CXC Chemokine Receptor 7 in chronic cardioprotection

Given the potential therapeutic utility of SDF-1α it has been the subject of several pre-clinical studies that have adopted a range of approaches to manipulate SDF-1α expression. For example, SDF-1α has been delivered by intracardiac injection immediately after AMI in mice and demonstrated activation of Akt within endothelial cells and cardiac myocytes, associated with improved cardiac function up to 28 days after infarction, increased VEGF, increased angiogenesis and reduced infarct size (Sasaki et al., 2007, Saxena et al., 2008). Continued expression of active SDF-1α (peak at 7 days) has been achieved by expression from adenovirus injected in the myocardium after infarction, which resulted in smaller infarct size, improved left ventricular parameters, less fibrosis and a greater density of cardiomyocytes and blood vessels in a rat model of STEMI (Tang et al., 2010). Other reported techniques include intracardiac injection of skeletal myoblasts overexpressing human SDF-1α (Deglurkar et al., 2006, Elmadbouh et al., 2007), MSCs overexpressing SDF-1α (Zhang, Mal, et al., 2007, Zhao et al., 2009), plasmid-based over-expression of SDF-1 (Sundararaman et al., 2011), use of a PEGylated fibrin patch for MSC transplantation (Zhang et al., 2007), and MSCs overexpressing IGF-1 to activate SDF-1α (Haider et al., 2008), all of which have conferred similar beneficial effects. Furthermore, some studies have utilised combinations of techniques. For example, Askari et al. administered cardiac fibroblasts over-expressing SDF-1α to the infarcted myocardium of a rat along with G-CSF and found improved stem cell homing, angiogenesis and cardiac function (Askari et al., 2003). Abbott et al. found that stem cells delivered to the coronary artery following AMI were only retained alongside adenovirus-mediated cardiac expression of SDF-1α, an effect that was abolished by AMD3100 (Abbott et al., 2004). Finally, Misao et al. administered G-CSF or vehicle 3 days after IR in rabbits and found increased serum SDF-1α, reduced infarct size, improved ejection fraction (EF) and improved end-diastolic dimensions in the G-CSF group, suggesting that G-CSF can increase SDF expression independently of its action on the SDF-CXCR4 association in bone marrow (Misao et al., 2006). This was abrogated by AMD3100 (Misao et al., 2006).

Likewise, studies have attempted to manipulate CXCR4 expression. Cheng et al. delivered MSCs overexpressing CXCR4 intravenously to rats 24 h after IR and found improved homing, better preservation of LV dimensions, reduced fibrosis and better LV function, as assessed by echocardiography (Cheng et al., 2008). Similarly, injection of cultured stem cells with CXCR4 specifically up-regulated by cultivation led to better angiogenesis (Shiba et al., 2009). Finally, conditional cardiomyocyte CXCR4 null mice have been used to investigate the requirement for CXCR4 signalling in stem cell-mediated repair following infarction (Dong et al., 2012). In this study, exogenous MSC engraftment was similar regardless of myocardial CXCR4 status, consistent with the hypothesis that, in these animals, the myocardium still produces SDF-1 and the MSCs still express CXCR4 (Dong et al., 2012). The authors noted abrogation of recovery of cardiac function following infarct in CXCR4 deficient mice, which may lend support to an acute role for SDF-1α, although infarct size was not measured acutely in this study (Dong et al., 2012).

**Fig. 2:**
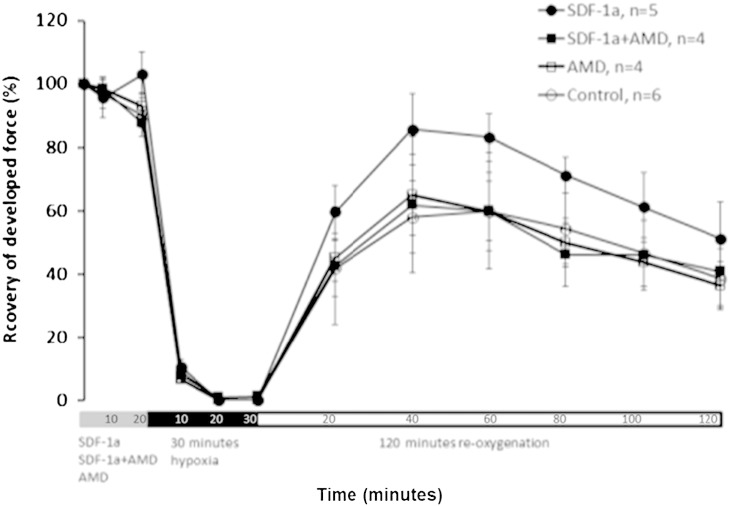
SDF-1α prior to ischaemia improves contractile recovery of rat heart papillary muscle that is isolated and subject to 30 min hypoxia and 2 h reoxygenation. Before hypoxia, the rat papillary muscle was perfused with 1) SDF-1 for 10 min; or 2) AMD3100 for 5 min then AMD3100 plus SDF-1 for 10 min, or 3) AMD3100 alone for 15 min. Control papillary muscle was untreated. Data expressed as mean ± SE. The functional recovery of muscle treated with SDF-1 alone was significantly improved (P < 0.05) (Davidson et al., 2013).

Despite these encouraging results enthusiasm is tempered by a lack of firm evidence for true myocardial regeneration, and it may be that the benefits described above are solely attributable to paracrine actions of SDF-1α (Ghadge et al., 2011). Furthermore, it is important to note that not all experimental data indicates a favourable role of SDF-1α–CXCR4 in AMI. For example, Pyo et al. found a negative inotropic effect of SDF-1 on murine papillary muscles and cardiac myocytes during calcium stimulation, an effect that was exacerbated by adenovirus-mediated over-expression of CXCR4 but attenuated by AMD3100 (Pyo et al., 2006). However, this data is contradicted by data from our laboratory showing that the functional recovery of muscle treated with SDF-1 was significantly improved relative to control muscle (see Fig. 2) (Davidson et al., 2013). Elsewhere, SDF-1α was injected into the peri-infarct zone of pigs that, consistent with previous studies, resulted in increased vessel density (Koch et al., 2006). However, there was no significant improvement in infarct size, myocardial perfusion or left ventricular function compared to controls (Koch et al., 2006). These studies may indicate a requirement for combination therapy to both stimulate stem cell mobilisation from bone marrow niches and homing to sites of injury. In addition, Koch et al. delivered only a single bolus of SDF-1, which could be expected to be rapidly proteolysed (Koch et al., 2006), and administered SDF-1 at 7 days while there is evidence that intravenous MSCs are only effective when given within 4 days (Maekawa et al., 2004).

Nevertheless, research into the role of the SDF-1α–CXCR4 axis has begun to be translated to the bedside. For example, Theiss et al. have confirmed in humans that mRNA of SDF-1 and HIF-1α, as well as stem cell factor and vascular cell adhesion molecule, are significantly higher in explanted heart tissue of patients with ischaemic cardiomyopathy versus dilated cardiomyopathy (Theiss et al., 2007). Penn's group have recently completed a Phase I study of plasmid-based endomyocardial SDF-1 delivery to 17 patients with symptomatic ischaemic cardiomyopathy and found improvements in 6-minute walk distance, New York Heart Association classification and quality of life (Penn et al., 2013). Despite these promising results, ejection fraction was non-significantly reduced in all patients and neither inflammation nor scar formation was recorded, which may be relevant with serial myocardial injection.

## 4 Acute cardioprotection

### 4.1 Ischaemic preconditioning

Given the role of SDF-1α–CXCR4/CXCR7 in myocardial repair and the involvement of signalling kinases known to be integral to ischaemic conditioning, namely the Erk1/2, PI3K-Akt, JAK-STAT and PKC signalling cascades (Deglurkar et al., 2006, Hausenloy and Yellon, 2008a), it has been hypothesised that SDF-1α may also be involved in the myocardial protection from IRI conferred by ischaemic conditioning. This is further supported by studies that have successfully used ischaemic preconditioning to up-regulate SDF-1α and improve stem cell engraftment after AMI. For example, Tang et al. applied a preconditioning stimulus directly to the heart in a murine model of AMI and demonstrated up-regulation of CXCR4 on cardiac progenitor cells, increased cardiac progenitor cell migration and recruitment, reduced infarct size and improved functional outcome, all of which were abolished by the addition of AMD3100 (Tang et al., 2009).

The pathophysiology of IRI is not fully understood, but proposed mechanisms include oxidative stress (Zweier, 1988), deranged calcium metabolism (Piper et al., 1998), rapid restoration of physiologic pH (Lemasters et al., 1996), inflammation (Vinten-Johansen, 2004), and deranged metabolism of glucose, insulin and potassium (Opie, 1970). However, manipulation of these mechanisms has largely failed to translate to benefit in clinical trials (Armstrong et al., 2007, Avkiran and Marber, 2002, Bar et al., 2006, Baran et al., 2001, Boden et al., 2000, Faxon et al., 2002, Granger et al., 2003, Hausenloy et al., 2010, Mehta et al., 2005, Mertens et al., 2006, Selker et al., 2012, Zeymer et al., 2001). The finding that the myocardium could be protected from lethal IRI by the application of multiple brief ischaemic episodes was first made by Murry et al., who found a 25% reduction in infarct size in dogs subjected to four 5 minute circumflex occlusions, each separated by 5 min of reperfusion, prior to sustained occlusion of the circumflex artery (Murry et al., 1986). They termed this phenomenon ischaemic preconditioning (IPreC) (Murry et al., 1986). However, despite promising experimental results, its clinical utility is limited by the necessity to intervene before the index ischaemia, which is evidently impossible to predict in STEMI. Further work by Zhao et al. investigated repetitive ischaemia applied in early reperfusion of the left anterior descending (LAD) territory in a canine model, and found a 14% reduction in infarct size (compared to 15% in IPreC in their model), a technique referred to as ischaemic post conditioning (IPostC) (Zhao et al., 2003). Several studies have investigated this approach in a clinical setting, with mixed results (Staat et al., 2005, Thibault et al., 2008, Lonborg et al., 2010, Sorensson et al., 2010, Freixa et al., 2012, Tarantini et al., 2012).

The mechanism of protection conferred by ischaemic conditioning, as it is currently understood, comprises extracellular autacoids acting on cardiomyocyte receptors in response to the conditioning stimulus and triggering protective intracellular signal transduction cascades (Ovize et al., 2010). These are thought to unite at the mitochondria, particularly the mitochondrial permeability transition pore (MPTP), to inhibit apoptosis and preserve cardiomyocyte viability (see Fig. 1) (Shanmuganathan et al., 2005).

Several endogenous extracellular factors are known to mitigate the deleterious effects of IRI and are thought to play a central role in ischaemic conditioning (Simpkin et al., 2007, Smith et al., 2010, Hausenloy and Yellon, 2013). They originate from a variety of sources, including cardiomyocytes, endothelium, smooth muscle cells, nerve endings, inflammatory cells, mast cells and macrophages, in response to the conditioning stimulus (Deussen et al., 1986, Dorge et al., 2002, Schulz et al., 2004, Faigle et al., 2008, Heusch et al., 2008). They are thought to have a number of actions, including reducing the activation of coronary vascular endothelium, reducing the production of pro-inflammatory cytokines and reactive oxygen species (ROS) and reducing adherence of neutrophils to the coronary artery, all of which contribute to ischaemic conditioning (Zhao et al., 2003, Halkos et al., 2004, Ovize et al., 2010). As described, these factors exert these effects on the intracellular effector by recruiting the same protein kinase signalling cascades that are thought to be activated by the SFD-1α–CXCR4/CXCR7 axis (Hausenloy and Yellon, 2004, Hausenloy and Yellon, 2007b, Ovize et al., 2010). The best defined of these are the ‘reperfusion injury salvage kinase’ (RISK) and ‘survivor activating factor enhancement’ (SAFE) pathways (Hausenloy and Yellon, 2007a, Boengler, Hilfiker-Kleiner, Drexler, Heusch and Schulz, 2008, Lacerda et al., 2009, Lecour, 2009).

The RISK pathway was first described by Yellon's group in recognition of the activation of PI3K-Akt pathway and p42/p44 Erk1/2 MAPK by myocardial reperfusion (Yellon and Baxter, 1999, Brar et al., 2000, Baxter et al., 2001, Jonassen et al., 2001, Hausenloy and Yellon, 2004). Pharmacological activation of this pathway has been shown to reduce infarct size by 40–50% at the time of reperfusion (Ovize et al., 2010). Importantly, several of the known protective endogenous factors, including insulin, insulin-like growth factor-1, bradykinin and adenosine, have been shown to protect against IRI by recruiting the RISK pathway (Hausenloy & Yellon, 2004). IPreC has also been shown to protect against lethal IRI by recruiting the RISK pathway (Tong et al., 2000, Fryer et al., 2001, Mocanu et al., 2002); specifically, the Akt1 isoform appears to be essential to IpreC as demonstrated in Akt1-deficient mice that were resistant to protection from IPreC (Kunuthur et al., 2012). Further, it has been shown that the RISK pathway is recruited equally by IPreC, IPostC and RIC, indicating that this signal transduction pathway may represent a common pathway in ischaemic conditioning (Hausenloy et al., 2003, Downey and Cohen, 2005, Hausenloy et al., 2005, Tamareille et al., 2011).

Lecour et al. described an alternative pathway, labelled the SAFE pathway, which activates JAK-STAT signalling (Lecour, 2009). In mice subjected to simulated ischaemia and reperfusion, IPostC reduced infarct size and increased phosphorylated (active) STAT3 (Boengler et al., 2008). In this study, administration of a specific JAK-2 inhibitor (AG-490) reduced phosphorylated STAT3 and abolished the beneficial effect of IPostC (Boengler et al., 2008). Likewise, cardiac-specific STAT3-deficient mice were not protected from IRI by IPostC (Boengler et al., 2008). Further, in a model of pharmacological preconditioning with tumour necrosis factor-α cardioprotection was not affected by inhibition of PI3K-Akt or Erk 1/2 MAPK, but was abolished by inhibition of STAT3 (Lecour et al., 2005). Likewise, the SAFE signalling pathway is also required for RIC (Tamareille et al., 2011). The interplay between RISK and SAFE pathways is not fully defined, however it has been shown that in ex vivo mouse hearts functional protection conferred by IPostC was not only abolished by JAK-STAT inhibition, but also that STAT3 inhibition decreased both functional STAT3 and Akt, suggesting that these signalling cascades are not entirely independent (Goodman et al., 2008). What is known is that both pathways converge on the mitochondria, particularly the MPTP, to affect cardioprotection.

Many of the pathological processes thought to mediate IRI, including oxidative stress, deranged calcium metabolism, and rapid recovery of physiologic pH, exert their effects at the mitochondria, resulting in cardiomyocyte death (Yellon & Hausenloy, 2007). Specifically, the MPTP, a voltage- and calcium-dependent channel in the inner mitochondrial membrane, is implicated (Ovize et al., 2010). This is closed in ischaemia due to acidosis, a high mitochondrial membrane potential, and high concentrations of Mg^2+^ and ADP (Di Lisa and Bernardi, 2009, Ovize et al., 2010). However, in reperfusion, the MPTP opens in response to binding of cyclophilin D (Cyp-D), which is potentiated by depolarization, increased mitochondrial Ca^2+^, inorganic phosphate and ROS, and restoration of normal pH (Griffiths and Halestrap, 1995, Di Lisa et al., 2001, Kim et al., 2006, Murata et al., 2001, Matsumoto-Ida et al., 2006, Ovize et al., 2010). Once open, the mitochondrial membrane rapidly dissipates, respiration becomes uncoupled, further elevating ROS formation, which establishes a vicious cycle of further MPTP opening and consequently loss of cell viability (Di Lisa and Bernardi, 2009, Ovize et al., 2010). Importantly, it is known that IPreC and IPostC antagonise MPTP opening and significantly limit infarct size in animal models of IR (Javadov et al., 2003, Hausenloy et al., 2004, Argaud, Gateau-Roesch, Muntean, et al., 2005, Argaud, Gateau-Roesch, Raisky, et al., 2005, Bopassa, Vandroux, Ovize and Ferrera, 2006, Zhao and Vinten-Johansen, 2006). Further, specific chemical inhibitors of MPTP, including NIM811 (Argaud, Gateau-Roesch, Muntean, et al., 2005, Argaud, Gateau-Roesch, Raisky, et al., 2005), cyclosporine A (Griffiths and Halestrap, 1993, Griffiths and Halestrap, 1995, Hausenloy et al., 2004, Argaud, Gateau-Roesch, Muntean, et al., 2005), and N-methyl-4-valine cyclosporine A (Hausenloy et al., 2004), have been shown to reduce MPTP opening, limit apoptosis, improve functional recovery, and limit infarct size. Likewise, transgenic Cyp-D-deficient mice subjected to simulated IR have been shown to have reduced infarct size (Baines et al., 2005, Nakagawa et al., 2005). The improvement in functional recovery conferred by cyclosporine A, which prevents Cyp-D binding (Ovize et al., 2010), has also been demonstrated in human atrial tissue (Shanmuganathan et al., 2005), has been successfully translated to a human pilot study of AMI (Piot et al., 2008), and a phase III trial is currently recruiting (“Cyclosporine & Prognosis in Acute Myocardial Infarction (MI) Patients (CIRCUS)”). Finally, it is notable that several studies have associated activation of the RISK and SAFE pathways with inhibition of MPTP opening, indicating it may represent the final common effector of ischaemic conditioning (Bopassa, Ferrera, Gateau-Roesch, Couture-Lepetit and Ovize, 2006, Davidson et al., 2006, Juhaszova et al., 2004, Smith et al., 2010, Zhao and Vinten-Johansen, 2006).

### 4.2 Stromal derived factor 1α and ischaemic conditioning

SDF-1α has been shown to be involved in myocardial protection from IRI conferred by ischaemic conditioning in a number of different models (Hu et al., 2007, Huang et al., 2011, Jang et al., 2012). Data from Yellon's group using ex vivo rat papillary muscle indicates that SDF-1α increases recovery of function of muscle subject to simulated IRI and can be blocked by AMD3100 (Fig. 2) (Davidson et al., 2013). Similarly, Huang et al. administered SDF-1 5 min before ischaemia in isolated mouse hearts subject to ischaemia–reperfusion in a model of pharmacological preconditioning (Huang et al., 2011). They found that SDF-1 significantly improved functional recovery, reduced markers of apoptosis and increased activation of STAT3, a central mediator of the SAFE pathway (Huang et al., 2011). These effects were abolished by the addition of AMD3100 (Huang et al., 2011). Interestingly, they did not see any increase in Akt or Erk1/2 phosphorylation, and no attenuation of protection with LY294002, an inhibitor of the Akt pathway (Huang et al., 2011). However, the inhibitors were given prior to ischaemia and not prior to reperfusion and hence it may be that Akt is still integral to mitigating reperfusion injury specifically. Jang et al. used an ex vivo Langendorff model of IR to show that SDF-1 infused from 10 min before reperfusion to 30 min afterwards reduced infarct size significantly more than that seen with IPreC and IPostC (Jang et al., 2012). They also saw an increase in Erk phosphorylation at 5 and 20 min after reperfusion, thereby implicating the RISK pathway in the mechanism (Jang et al., 2012). Hu et al. demonstrated significantly increased SDF-1α released from isolated cardiac myocytes following hypoxia and reoxygenation that resulted in increased phosphorylation of both Erk1/2 and Akt, less lactate dehydrogenase release and less apoptosis (Hu et al., 2007). Pre-treatment with AMD3100 abolished the effect on myocyte survival (Hu et al., 2007). In vivo, they demonstrated pharmacological preconditioning with SDF-1α infused into the left ventricular cavity significantly reduced infarct size, which was abrogated by AMD3100 (Hu et al., 2007). Conversely, Chen et al. used adenovirus-mediated over-expression of CXCR4 administered 7 days prior to IR in rats and found significantly increased scar size, worse fractional shortening, increased inflammatory cell infiltration, increased cardiac myocyte apoptosis and more left ventricular hypertrophy at 24 h (Chen et al., 2010). These effects were attenuated by AMD3100, and it is clear that the window for protection from IRI requires careful evaluation (Chen et al., 2010).

### 4.3 Remote ischaemic conditioning

Despite promising results for mechanical IPreC and IPostC, the common problem is that both mandate an invasive approach to cardioprotection that may be both impractical and even harmful, as they increase the risk of procedural complications, including access site complications, coronary artery dissection or perforation, arrhythmias and stroke. In response to these concerns RIC has emerged as an exciting potential alternative, whereby brief cycles of ischaemia and reperfusion are applied to an organ or tissue remote from the heart. This was first shown to be protective by Przyklenk et al., 1993, who applied four episodes of 5 minute circumflex occlusion separated by 5 min of reperfusion, before 1 h of sustained LAD occlusion and reperfusion for 4.5 h in a canine model (Przyklenk et al., 1993). They found a 10% reduction in infarct size in the circumflex preconditioned dogs (Przyklenk et al., 1993). This has been developed by others who showed similar cardioprotective effects after applying a preconditioning stimulus to other remote organs and tissues, including the kidneys and skeletal muscle (Mcclanahan et al., 1993, Birnbaum et al., 1997), and using a model of remote ischaemic postconditioning (Andreka et al., 2007). More recently, it has been demonstrated that the non-invasive application of brief cycles of ischaemia and reperfusion to a limb using a tourniquet has the same effect (Oxman et al., 1997, Kharbanda et al., 2002), a finding which has greatly accelerated the rate of clinical trials demonstrating RIC to be protective in a variety of clinical settings (Candilio et al., 2013). These include prior to coronary artery bypass surgery (CABG) (Gunaydin et al., 2000, Cheung et al., 2006, Hausenloy et al., 2007, Venugopal et al., 2009, Thielmann et al., 2010, Hausenloy et al., 2012, Thielmann et al., 2013), elective abdominal aortic aneurysm repair (Ali et al., 2007, Walsh et al., 2009), elective cervical decompression surgery (Hu et al., 2010), elective PCI (Hoole et al., 2009), and in PPCI for STEMI (Kerendi et al., 2005, Schmidt et al., 2007, Botker et al., 2010). For patients suffering STEMI, Botker et al. randomised patients to receive PPCI with or without a pre-hospital RIC protocol. The primary endpoint of improved myocardial salvage index at 30 days, measured by myocardial perfusion imaging, was met (Botker et al., 2010). Importantly, studies have demonstrated similar effects with RIC applied before (Gunaydin et al., 2000, Hausenloy et al., 2007, Rentoukas et al., 2010), during (Botker et al., 2010), and after the index ischaemia (Kerendi et al., 2005, Andreka et al., 2007), thereby improving its clinical utility and potential to mitigate IRI in patients.

It is suggested that the protective effect of RIC may be due to a humoral factor(s), which may be one or a combination of the factors described above, or a novel molecule(s), which are carried by the blood from the transiently ischaemic limb to the remote target organ where they activate endogenous pro-survival signalling pathways. Evidence for this comes from studies wherein the cardioprotective effect of RIC applied to the lower limb is abrogated by occlusion of the femoral vein (Lim et al., 2010). Further, infarct size is significantly reduced when the effluent from an isolated perfused heart that is preconditioned is used to perfuse a second isolated heart prior to index ischaemia (Dickson et al., 1999, Leung et al., 2013). Interestingly, there also appears to be a neural component to RIC whereby severing the femoral and sciatic nerve in an in vivo mouse model of IR abolishes the protection conferred by RIC, although the relationship between neural and humoral components of this phenomenon is debated (Lim et al., 2010).

### 4.4 Stromal derived factor 1α and remote ischaemic conditioning

For the reasons described, SDF-1α is a prime candidate for a role in RIC. Although only a limited number of studies have investigated the possible role of SDF-1α–CXCR4 in RIC, it so far satisfies the criteria for an endogenous mediator defined by the Working Group of Cellular Biology of the Heart of the European Society of Cardiology (Ovize et al., 2010); namely that RIC can be abrogated by specific receptor blockade or inhibition of the mediator's production and that RIC can be mimicked by exogenous administration of the mediator. Jiang et al. used remote ischaemic postconditioning to improve the retention of improved MSCs in a murine model of myocardial infarction (Jiang et al., 2013). They found increased serum and myocardial SDF-1α, significantly increased MSC retention in the myocardium, and improved cardiac function at 1 month, all of which was abrogated by administration of anti-rat CXCR4 polyclonal antibody as a single intraperitoneal injection after the RIC procedure (Jiang et al., 2013). Kamota et al. conferred RIC using cyclical occlusion of the abdominal aorta in mice prior to IR of the LAD territory (Kamota et al., 2009). They found increased VEGF and SDF-1α acutely (1 and 3 h) and significantly increased CD34^+^ stem cells in the peripheral blood at 12 and 24 h (Kamota et al., 2009). Both phases of protection independently resulted in improved LV dimensions and function, and less apoptosis (Kamota et al., 2009). Interestingly, blocking recruitment of bone marrow stem cells only abrogated cardioprotection in the late phase in their model, which all suggests that SDF-1α has the ability to potentially repair myocardial damage, by directing the homing of stem cells from the bone marrow to the site of damage, in combination with its potential to directly protect the myocardium from IRI via the pathways described above. Most recently, Davidson et al. demonstrated the involvement of SDF-1α in acute RIC by showing that RIC significantly reduced infarct size, an effect which was blocked by AMD3100 (Davidson et al., 2013). The also confirmed that SDF-1α was elevated in the plasma of rats subjected to hind-limb RIC (Davidson et al., 2013). Improved functional recovery in isolated rat cardiac papillary muscle subjected to simulated IR after RIC was also blocked by AMD3100 and in this model significant functional recovery was also seen with pharmacological preconditioning with SDF-1α (Davidson et al., 2013).

## 5 Dipeptidyl peptidase-4 inhibitors

Despite these exciting results, an important drawback is the relatively short plasma half-life of SDF-1α, which might limit its therapeutic utility (Valenzuela-Fernandez et al., 2002, Segers et al., 2007). However, bioengineered SDF-1 that is resistant to cleavage by DPP-4 and MMP-2 has been associated with improved stem cell homing, angiogenesis and ejection fraction (Segers et al., 2007). Another approach to extracting the maximum potential from SDF-1α relates to its potential manipulation by a new class of anti-diabetic drugs. DPP-4 inhibitors such as Sitagliptin, Vildagliptin, Alogliptin and Saxagliptin, have been designed to prevent the breakdown of the incretin glucagon-like peptide 1 (GLP-1) by inhibiting the protease DPP-4 thereby increasing insulin and lowering glucose (Ravassa et al., 2012). Active SDF-1α is also cleaved by DPP-4 and thus, similar to GLP-1, DPP-4 inhibition increases the half-life of SDF-1α by preventing its degradation (Crump et al., 1997, Zaruba et al., 2009). DPP-4 is found on many of the same cell types as CXCR4, including B and T lymphocytes, endothelial cells and CD34^+^ HPCs, as well as being present in plasma (Christopherson et al., 2003, Zaruba and Franz, 2010).

Several studies have attempted to exploit this proteolytic mechanism. Christopherson et al. first showed that DPP-4 inhibition increased stem cell homing to bone marrow (Christopherson et al., 2004), following which Zaruba et al. combined DPP-4 inhibition using Diprotin A with G-CSF-mediated stem cell mobilisation in a murine model of myocardial infarction (Zaruba et al., 2009). They found decreased DPP-4 activity, which was associated with the stabilisation of active SDF-1α (Zaruba et al., 2009). This consequently increased CXCR4^+^ EPCs homing (an effect that was abrogated by administration of AMD3100), reduced cardiac remodelling and apoptosis, and improved EF and survival (Zaruba et al., 2009). This approach was recently tested in a phase III clinical trial using Sitagliptin (Safety and efficacy of SITAgliptin plus Granulocyte-colony-stimulating factor in patients suffering from Acute Myocardial Infarction, SITAGRAMI). They randomised patients to either G-CSF and Sitagliptin or placebo after PPCI for AMI in a multi-centre, double-blind design. The primary endpoint of improved combined global left and right ventricular ejection fraction as assessed by magnetic resonance imaging was not met, and while there was a trend towards reduced major adverse cardiac events this was not significant (“Safety and efficacy of SITAgliptin plus Granulocyte-colony stimulating factor in patients suffering from Acute Myocardial Infarction — SITAGRAMI trial”). This may be explained by the inclusion of only 21% of patients with left ventricular ejection fraction below 50%, thereby obfuscating any potential benefit of this therapy.

Similarly, two large multicentre clinical trials have recently failed to demonstrate a benefit of DPP-4 inhibitors on cardiovascular outcomes in patients with type 2 diabetes at high risk for cardiovascular events (Scirica et al., 2013, White et al., 2013). For example, SAVOR-TIMI 53 compared Saxagliptin and placebo in 16,492 patients with a history of, or at risk for, cardiovascular events (Scirica et al., 2013). After median follow-up of 2.1 years they found no significant difference in the primary endpoint of cardiovascular death, myocardial infarction or stroke. Similarly, EXAMINE compared Alogliptin with placebo in 5380 patients with type 2 diabetes and recent acute coronary syndrome (ACS) over median follow-up of 18 months and found no significant difference in the primary endpoint of cardiovascular death, nonfatal myocardial infarction or nonfatal stroke (White et al., 2013). That these important studies failed to show any long-term benefit of DPP-4 inhibitors is disappointing, but may have several explanations. Patients in SAVOR-TIMI 53 had not necessarily had a recent ACS or other hypoxic stimulus to SDF-1α production and it may be the case that, as in the preclinical studies described, a combination of SDF-1α up-regulation and DPP-4 inhibition is necessary. In EXAMINE, although a recent ACS was an inclusion criteria, re-admissions with heart failure were not recorded.

It is equally important to note that these studies did not specifically examine any acute role of DPP-4 inhibition, specifically on IRI. Indeed, relatively few groups have done so. Kanki et al. developed a bioengineered form of SDF-1 resistant to MMP-2 and DPP-4 (SSDF-1(S4V)), which they injected into the LV cavity after reperfusion in a rat model of IR (Kanki et al., 2011). This resulted in improved retention in the ischaemic myocardium (Kanki et al., 2011). They also found improved function and capillary density, although this was several weeks after the initial insult making it difficult to separate the relative contribution of acute cardioprotection and possible cardiac regeneration. It has also been demonstrated that the new anti-diabetic agents described above have direct cardioprotective effects by preventing cell death and limiting infarct size in ex vivo models (Hausenloy et al., 2013). Hausenloy et al. pretreated control and diabetic rats with DPP-4 inhibitors Vildagliptin or Sitagliptin and found a significant reduction in infarct size (Hausenloy et al., 2013). Interestingly, they found this to be dependent on elevated glucose and inhibited by Exendin, a GLP-1 receptor antagonist (Hausenloy et al., 2013). However, the contribution, if any, of SDF-1–CXCR4 was not elucidated.

## 6 Conclusions

Reperfusion injury makes an important contribution to myocardial injury and poor clinical outcomes after a lethal ischaemic insult in animal models, although there remains a paucity of data from clinical studies. Ischaemic conditioning has emerged as a powerful protective phenomenon and has translated to the clinical application of remote ischaemic conditioning, which is a clinically feasible, non-invasive, cost-effective therapeutic intervention that has been shown to mitigate acute myocardial injury in preliminary studies. This has heralded a hunt for the potential mechanism, which is thought to be due to a humoral factor(s) carried from the preconditioned organ or tissue to the heart, where endogenous pro-survival signalling pathways are activated. SDF-1α confers protection against myocardial ischaemia–reperfusion injury via the same signalling pathways implicated in ischaemic conditioning, namely the RISK and SAFE pathways. It is also central to the mobilisation and migration of stem cells and has been used to target them to sites of ischaemic injury. SDF-1α therefore potentially has pleiotropic effects on ischaemic myocardium: directly protecting via intracellular pro-survival signal transduction pathways while activating stem cell mobilisation and gradient-guided homing to augment myocardial recovery. One potential avenue for translating these findings to the bedside is with regard to the potentially significant role of SDF-1α in the mechanism of action of the anti-diabetic DPP-4 inhibitors to protect the myocardium from lethal reperfusion injury. Finding the factor(s) responsible for RIC is both novel and paramount for maximising its potential in cardiac patients. Further work should focus on clarifying the role of SDF-1α–CXCR4 in cardioprotection and whether this can be mimicked using DPP-4 inhibitors acutely.
